# Carrying what came after: post-migration difficulties and depression among refugees and asylum seekers

**DOI:** 10.1186/s13031-025-00728-3

**Published:** 2025-12-02

**Authors:** Arwin Nemani, Schahryar Kananian, Annabelle Starck, Ahlke Kip, Dana Churbaji, Nexhmedin Morina, Ricarda Nater-Mewes, Cornelia Weise, Hannah Preiß, Thomas Ehring, Ulrich Stangier

**Affiliations:** 1 Department of Psychology, Goethe University Frankfurt, Varrentrappstraße 40-42, 60486 Frankfurt, Germany; 2 Medical Psychology and Medical Sociology, Faculty of Medicine, University of Freiburg, Freiburg, Germany; 3 Institute of Psychology, University of Münster, Münster, Germany; 4 Institute of Psychology, Clinical Psychology and Psychotherapy, University of Klagenfurt, Universitätsstraße 65-67, Klagenfurt, 9020 Austria; 5 Dept. of Psychology, Clinical Psycho- logy and Behavioral Health Technology, Friedrich-Alexander-Universität Erlangen-Nürnberg, Nägelsbachstr. 49b, 91052 Erlangen, Germany; 6 Department of Psychology, LMU Munich, Leopoldstr. 13, 80802 Munich, Germany

**Keywords:** Post-migration stressors, Refugee mental health, Latent profile analysis, Depressive symptoms, Network analysis

## Abstract

**Background:**

Refugees and asylum seekers encounter numerous post-migration living difficulties (PMLDs) that can substantially affect their mental health. However, the role of PMLDs remains insufficiently explored, particularly in clinical refugee populations. This study aimed to identify subgroups based on patterns of PMLD by examining their relationship with depressive symptoms and determining which stressors function as key bridges.

**Methods:**

This study reports a secondary analysis of baseline data from the ReTreat trial. Data were collected from 141 refugees and asylum seekers enrolled in a multicentre randomized controlled trial of a culturally adapted CBT program in Germany. Participants completed measures of depressive symptoms (PHQ-9) and post-migration stressors (27-item checklist). Latent Profile Analysis (LPA) was used to identify distinct burden profiles. Exploratory Factor Analysis (EFA) examined the dimensionality of PMLDs. Network analysis was conducted to investigate symptom–stressor connectivity.

**Results:**

Three latent profiles emerged: Class 1 showed elevated distress across all domains; Class 2 was characterized by family separation and homesickness; and Class 3 exhibited minimal post-migration stress. EFA of PMLDS supported a four-factor solution: institutional/legal stressors, structural hardship, health/service access, and emotional/family-related strain. Depressive symptoms differed significantly across profiles, with highest scores in the high burden group (Class 1). Network analysis identified institutional/legal and emotional/family-related stressors as central bridge nodes linking PMLDs to depressive symptoms.

**Conclusions:**

PMLDs are multidimensional and heterogeneously distributed among forcibly displaced individuals. Legal insecurity and emotional strain are particularly influential in connecting environmental hardship to depressive symptoms.

**Trial registration:**

This study uses baseline data from a registered randomized controlled trial (DRKS00021536).

**Supplementary Information:**

The online version contains supplementary material available at 10.1186/s13031-025-00728-3.

## Background

 The mental health of forcibly displaced individuals remains a pressing concern across host countries worldwide [1]. Refugees and asylum seekers from conflict-affected regions such as Afghanistan, Syria, or Ukraine often endure cumulative and complex adversities before, during, and after flight. While considerable research has focused on the impact of pre-migration trauma, including war and persecution [2, 3], increasing evidence suggests that post-migration stressors may exert an equally, if not more, enduring influence on psychological health [4–6]. Importantly, these effects have been documented not only among refugees with recognized status, but also among asylum seekers who often face even greater mental health risks due to ongoing legal insecurity and restricted access to services [3].

Post-migration living difficulties (PMLDs) refer to chronic and often systemic stressors encountered after arrival in a host country. These include legal insecurity (e.g., pending asylum claims), economic hardship, limited access to healthcare and employment, social exclusion, and separation from family [1]. Such stressors are not only persistent but also deeply embedded within institutional and societal structures, often extending the emotional burden of forced migration [7, 8]. Depression is among the most prevalent mental health outcomes in this context, commonly maintained or exacerbated by the cumulative impact of these post-migration adversities [9, 10].

Although early models have emphasized trauma exposure as the primary predictor of mental ill-health in refugees, studies have consistently found that traumatic events account for a relatively small proportion of the variance in mental health outcomes [11, 12]. This has led to the development of ecological and interactional models that recognize the critical role of post-migration environments in shaping long-term mental health trajectories [6].

While this role is increasingly recognized, many studies treat PMLDs as a cumulative score, implying equal weight and impact across diverse stressors [13, 14]. However, not all PMLDs contribute equally to psychological distress. Certain stressors—such as asylum insecurity or family separation—may have disproportionate effects on depressive symptoms [15]. Variable-centered analyses (e.g., factor analysis) have revealed meaningful clusters of post-migration stressors [16, 17], yet these approaches assume population homogeneity and cannot capture subgroup-specific patterns of risk.

To address this limitation, person-centered approaches such as Latent Profile Analysis (LPA) offer a nuanced alternative. These methods identify subgroups of individuals who experience similar patterns of stressors, allowing researchers to detect distinct profiles of vulnerability and resilience [18, 19]. While many earlier studies applied LPA primarily to trauma symptom profiles, Byrow et al. [19] reported that such techniques can be successfully used to classify resettled refugees based on their post-migration stressor exposure. However, their findings were based on a community-based sample of resettled individuals with relatively secure legal status and integration into the host society.

The present study builds on and extends this work by examining a clinically referred refugee population with elevated mental health needs, including many who face legal insecurity, collective accommodation, and greater spatial or cultural distance from their country of origin. Furthermore, we combine LPA with factor-analytic and network-based approaches to explore not only subgroup structures, but also the central pathways through which specific PMLD domains relate to depressive symptoms. This integrated strategy aims to yield a more comprehensive understanding of how systemic stressors shape psychological outcomes in displaced populations.

Building on this foundation, the present study used LPA and network analytic techniques to further examine how patterns of PMLDs manifest within a clinical refugee sample. Specifically, we addressed three research questions: (1) What distinct patterns of PMLDs can be identified among refugees and asylum seekers? (2) Do these patterns correspond to distinct risk profiles for depressive symptoms? (3) Which specific stressors serve as key bridges between environmental burden and psychological distress?

## Methods

### Sample

The present study is a secondary analysis of baseline data from ReTreat study, a multicentre randomized controlled trial (RCT) evaluating the efficacy of culturally adapted cognitive behavioural group therapy (CA-CBT+) for refugees and asylum seekers with mental disorders in Germany [20]. The sample included both refugees (recognized status) and asylum seekers (pending claims); primary analyses pooled both groups due to the exploratory scope and limited power for status-stratified models. The sample size was determined a priori in the ReTreat study protocol [20], which calculated that 138 participants were required to detect the main treatment effects of the RCT (accounting for dropouts). The present analyses are secondary and exploratory, and no additional power calculation was conducted. The achieved sample (*N* = 141) exceeded the planned number. The trial was conducted across four sites—Frankfurt, Marburg, Munich, and Münster—and targeted a wide spectrum of psychopathological symptoms using a transdiagnostic intervention framework. Participants were recruited through outpatient clinics, psychosocial centres, and partner organizations serving asylum seekers and refugees. Eligible participants were adult refugees and asylum seekers (aged 18–65 years) with a background of forced displacement who screened above clinical thresholds on the General Health Questionnaire (GHQ-28 >= 11) and met diagnostic criteria for at least one DSM-5 disorder according to the Mini-International Neuropsychiatric Interview (M.I.N.I.). Exclusion criteria included current substance use disorders, acute suicidality, psychosis, or ongoing psychotherapy. Screening and assessments were conducted with culturally adapted instruments in participants’ native languages (e.g., Farsi, Arabic, Russian), supported by trained bilingual staff. All procedures were approved by the Ethics Commission of the German Psychological Society (Ref: StangierUlrich2019-1018VA), and participants gave written informed consent.

## Measures

Depressive symptoms were assessed using the 9-item Patient Health Questionnaire (PHQ-9) [21], a validated self-report measure of depression severity with good psychometric properties across diverse cultural contexts [22]. Each item reflects a DSM-5 symptom criterion and is rated on a 4-point Likert scale (0 = “not at all” to 3 = “nearly every day”). Internal consistency in the present sample was good, with Cronbach’s alpha = 0.89 and McDonald’s omega = 0.92.

Post-migration living difficulties (PMLDs) were measured using a culturally adapted version of the Post-Migration Living Difficulties Checklist (PMLDC) [23], consisting of 27 items. Respondents indicated the degree of burden caused by each stressor over the past 12 months on a 5-point scale (0 = “not a problem” to 4 = “a very serious problem”). Internal consistency was similarly high (Cronbach’s alpha = 0.90, McDonald’s omega = 0.92).

Mental health diagnoses were established using the Mini-International Neuropsychiatric Interview (M.I.N.I. 7.0) [24], adapted for DSM-5 criteria. The interview was administered by trained clinicians and bilingual assistants. The M.I.N.I. is a structured diagnostic interview that assesses major psychiatric disorders and has demonstrated good reliability and validity across cultures. Interviews were conducted by trained clinicians or advanced clinical psychology students, supported by bilingual assistants. To ensure cultural and linguistic appropriateness, assessments were administered in participants’ most commonly spoken native languages (e.g., Farsi, Arabic, Dari). For instruments without validated translations, standard translation and back-translation procedures were applied by independent native speakers, and discrepancies were resolved by consensus. When necessary, interviews were carried out with interpreter assistance.

### Data analysis

All statistical analyses were conducted using R (version 4.5.0; R Core Team, 2025), employing established tools for latent modeling and psychological network estimation. The analyses were exploratory and informed by contemporary approaches to dimensionality and symptom interconnectivity in psychopathology [25, 26]. In the dataset, a total of 501 values (12.69%) were missing across the 27 PMLD items, and 183 values (12.98%) were missing across the nine PHQ-9 depression items. Missing data were handled using multiple imputation via predictive mean matching (PMM), as implemented in the mice package [27].

To address our research questions, we conducted three sets of analyses in the following order:

#### (1) Latent profile analysis (LPA)

To answer RQ1, we applied LPA to individual PMLD item responses to identify distinct subgroups of refugees and asylum seekers with similar stressor patterns. Model selection was guided by fit indices including the Bayesian Information Criterion (BIC), entropy values, and theoretical interpretability [28, 29].

To examine differences between latent classes, we compared sociodemographic variables and clinical characteristics across groups. One-way ANOVAs with Tukey’s HSD post-hoc tests were conducted for continuous variables, and χ²-tests were used for categorical variables.

#### (2) Exploratory factor analysis (EFA)

To answer RQ2, we conducted an EFA using maximum likelihood extraction with oblimin rotation to examine the latent structure of PMLDs and identify meaningful stressor domains. The number of factors was determined via parallel analysis [30] and theoretical interpretability. To assess the relationship between post-migration stressors and depressive symptoms, we conducted linear regression analyses with total PMLD scores predicting PHQ-9 depression severity. In addition, exploratory Pearson correlations between PMLD and PHQ-9 scores were calculated separately within each latent class.

#### (3) Network analysis

To answer RQ3, we estimated a regularized partial correlation network including the derived PMLD factors and the nine PHQ-9 items. The EBICglasso algorithm [31] was used to obtain a sparse and stable structure. Bridge strength centrality was calculated using the *networktools* package [32] to identify nodes linking stressors and depressive symptoms. Accuracy and robustness were assessed via bootstrapped confidence intervals (1,000 iterations) for edge weights and node stability, applying recommended interpretability thresholds for centrality stability (CS ≥ 0.50) [31]. Network visualizations were created with the *qgraph* package [33]. In line with best practices, no p-values were reported for individual edges [34].

## Results

### Sample characteristic

A total sample of 141 participants (75.2% male, mean age = 33.1 years, SD = 10.6) were recruited across four sites in Germany (Frankfurt, Marburg, Munich, and Münster). Participants originated primarily from Afghanistan (52.1%), followed by Syria (17.1%), Iran (14.3%), and others. Educational attainment varied, with 27.2% holding a university degree and 22.1% reporting no formal education. On average, participants had spent 51.2 months in Germany (SD = 48.7) and 9.7 months in transit to Germany (SD = 24.8). All baseline characteristics are summarized in Table 1. Demographic variables did not significantly predict overall PMLD scores, *F*(5, 125) = 1.09, *p* =.37, *R*² = 0.04. No individual demographic predictor, including gender, reached significance (ps > 0.20).

**Table 1:** Demographic and baseline characteristics in the study population

**Characteristic**	**Sample**, N = 141
**Sex (male/female), n/N (%)**	
Women	35/141 (24.8%)
Men	106/141 (75.2%)
**Age (years)**	
Mean (±SD)	33.1 (±10.6)
**Residential situation, n/N (%)**	
Own Apartment	57/138 (41.3%)
Initial Reception Center	11/138 (8%)
Shared Housing	63/138 (45.7%)
With Family/Friends	5/138 (3.6%)
Other Residential situation	2/138 (1.4%)
Unknown	3
**Asylum status/residence permit, n/N (%)**	
Asylum-seeking status	46/139 (33.3%)
Residence permit	47/139 (34.1%)
Tolerated status	28/139 (19.6%)
Other	18/139 (13%)
Unknown	2
**Nationality, n/N (%)**	
Afghanistan	73/140 (52.1%)
Iran	20/140 (14.3%)
Syria	24/140 (17.1%)
Irak	5/140 (3.6%)
Ukraine	10/140 (7.1%)
Other Arabic country	8/140 (5.7%)
Unknown	1
**Socioeconomic situation, n/N (%)**	
Much better	6/139 (4.3%)
better	33/139 (23.6%)
Same	30/139 (21.4%)
Worse	44/139 (31.4%)
Much worse	26/139 (18.6%)
Unknown	2
**School years**	
Mean (±SD)	10.1 (±3.3)
Unknown (n)	4
**Stay in Germany (months)**	
Mean (±SD)	51.2 (±48.7)
Unknown (n)	2
**Transit time to Germany (months)**	
Mean (±SD)	9.7 (±24.8)
Unknown (n)	5

Percentages are based on valid responses only (excluding unknowns). “Unknown” refers to participants for whom data were not available. Bolded subheadings group related variables

### Latent profile analysis

**Fig. 1:**
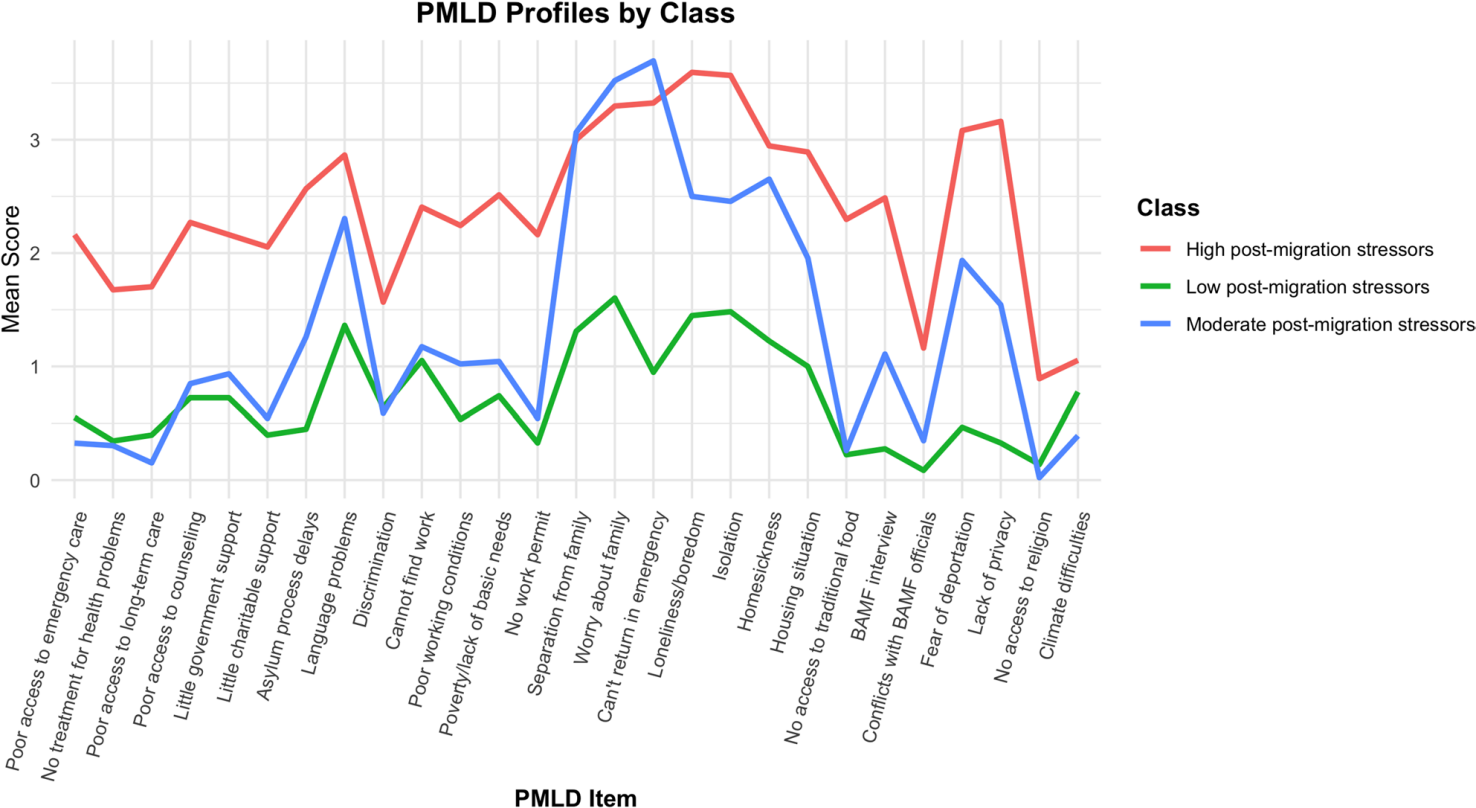
Profiles of post-migration stress based on latent profile analysis. Mean scores across 27 post-migration living difficulty (PMLD) items for the three classes identified by latent profile analysis (LPA): high (red), moderate (blue), and low (green) stressor groups

Latent Profile Analysis (LPA) based on 27 PMLD items revealed a 3-class solution with good model fit (BIC = 10,245.41; Entropy = 0.95). Although the 4-class solution showed a slightly lower BIC, the inclusion of a small subgroup (< 15%) indicated possible overfitting. The selected model identified three distinct profiles of post-migration stress (Fig. 1).

Class 1 – High post-migration stressors (*n* = 37) reported the highest levels of distress across nearly all PMLD domains, with particularly elevated means for loneliness (*M* = 3.59), isolation (*M* = 3.57), not to return in emergency to family (*M* = 3.32), Worry about the family (*M* = 3.21) and lack of privacy (*M* = 3.16).

Class 2 – Moderate post-migration stressors (predominantly family-related) (*n* = 46) showed a similar burden in family-related items, such as worry about family members (*M* = 3.52), not being able to return (*M* = 3.70), and separation from family (*M* = 3.07). In contrast, practical stressors such as lack of access to healthcare (*M* = 0.33) or poverty (*M* = 1.04) were notably lower.

Class 3 – Low post-migration stressors (*n* = 58) reported the lowest PMLD scores overall, with means below 1 on most items, including fear of deportation (*M* = 0.33) and discrimination (M = 0.64), indicating relatively few post-migration stressors. Nonetheless, even in this group, some relational and social difficulties were reported above minimal levels, particularly worry about family (M = 1.60), isolation (M = 1.48), loneliness (M = 1.45), language problems (M = 1.36), separation from family (M = 1.31), and homesickness (M = 1.22). Difficulties in finding work were also present, though less pronounced (M = 1.05). Taken together, the results highlight not only differing levels of post-migration stress across subgroups, but also distinct qualitative patterns.

## Class differences

The latent profiles differed not only in levels and patterns of post-migration living difficulties (PMLDs), but also in depressive symptoms and selected sociodemographic characteristics. Additional class characteristics are summarized in Table 2.

Class 1 – High post-migration stressors (*n* = 37) reported the highest overall burden of post-migration living difficulties (PMLD; M = 65.89, SD = 12.27), significantly higher than both other groups (*p* <.001). Participants were predominantly male (83.8%) and had low educational attainment: 30.6% reported no school degree, while 25.0% held a university degree. The mean age was 32.97 years (SD = 10.47), and they had completed an average of 9.24 years of education (SD = 4.46). Most were from Afghanistan (48.6%) or Syria (18.9%), and had resided in Germany for 43.28 months on average (SD = 25.63).

Class 2 – Moderate post-migration stressors (predominantly family-related) (*n* = 46) showed a moderate PMLD burden (M = 37.39, SD = 9.34), also significantly different from both other classes (*p* <.001). This group had the highest average duration of transit migration (M = 14.75 months, SD = 36.70), and reported the lowest educational attainment (M = 8.05 school years, SD = 4.66). A third had no formal education (31.1%), and 17.8% held a university degree. The group was 69.6% male, with a majority from Afghanistan (65.2%) and Iran (13%).

Class 3 – Low post-migration stressors (*n* = 58) reported the lowest total PMLD burden (M = 20.47, SD = 8.07), significantly below both Class 1 and 2 (*p* <.001). This group also reported significantly fewer depressive symptoms (PHQ-9 M = 9.21, SD = 6.76) compared to Class 1 (*p* =.027) and Class 2 (*p* <.001). Participants had the highest average years of schooling (M = 10.14, SD = 3.61, *p* =.050) and were more often university-educated (36.4%) with only 9.1% lacking formal qualifications. They had also spent the longest time in Germany (M = 59.74 months, SD = 67.07), though this difference was not statistically significant (*p* =.214). Their countries of origin were more diverse, including higher proportions from Syria (24.1%) and Ukraine (12.1%) alongside Afghanistan (41.4%).

The observed differences between classes regarding gender, educational qualification, and country of origin were not statistically significant (gender: *p* =.32; education: *p* =.11; origin: *p* >.05, all comparisons).

**Table 2:**
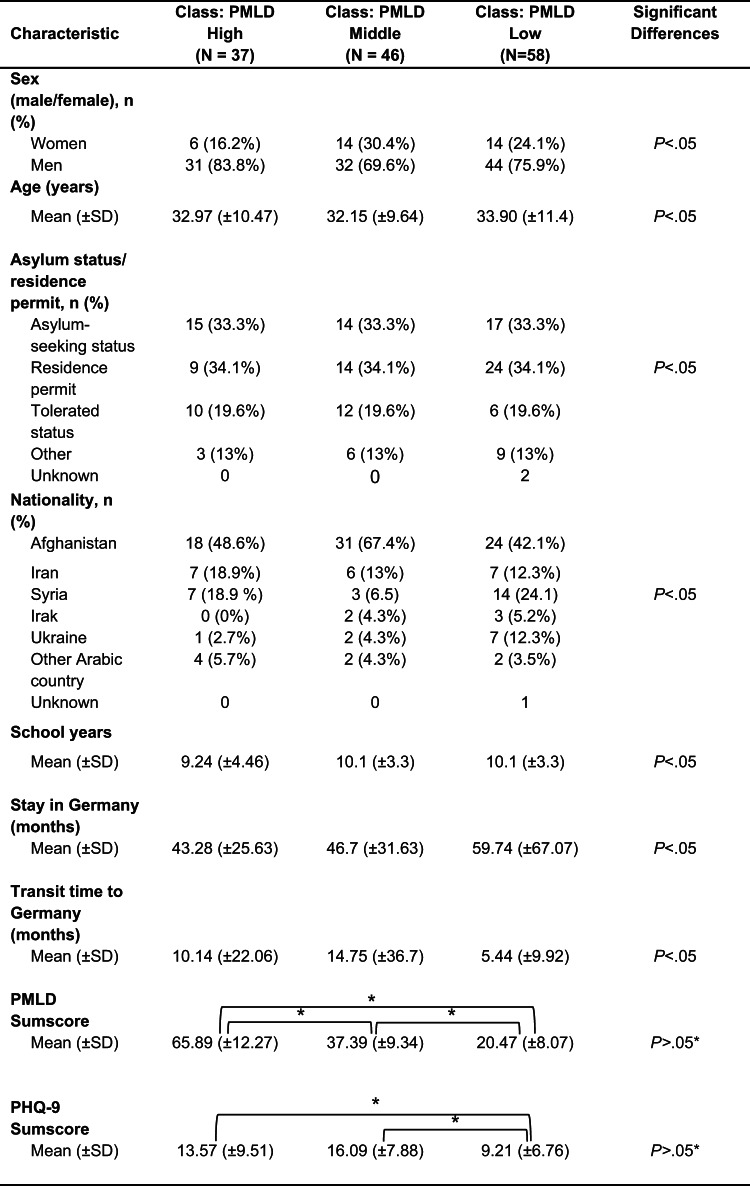
Demographic and baseline characteristics in study population between 3 Classes

Differences marked with an asterisk (*) indicate statistically significant group differences. Mean differences between groups were assessed using Tukey’s Honestly Significant Difference (Tukey HSD) test following a one-way ANOVA. The Tukey HSD results for the total PMLD score showed significant differences between all groups (adjusted p-values <.001). Frequency differences across groups were analyzed using Chi-square tests. Overall group differences in means were examined with one-way ANOVA

## Exploratory factor analysis of post-migration living difficulties (PMLD)

To identify the latent structure of post-migration stressors, an exploratory factor analysis (EFA) was conducted on the 27 PMLD items using maximum likelihood extraction with oblimin rotation. The data met psychometric assumptions, with a Kaiser-Meyer-Olkin (KMO) value of 0.82 indicating meritorious sampling adequacy, and Bartlett’s test of sphericity confirming sufficient intercorrelation (χ²(351) = 2124.09, *p* <.001).

Parallel analysis suggested five factors, but a four-factor solution was retained based on theoretical coherence and model interpretability. This model accounted for 49% of the total variance (15%, 13%, 12%, and 10% across the four factors). Model fit was moderate (RMSR = 0.06; RMSEA = 0.093, 90% CI [0.084, 0.104]; TLI = 0.749), and factor correlations ranged from *r* =.13 to 0.45.

The first factor reflected institutional and legal stressors, including fear of deportation, delays in the asylum process, experiences of discrimination, and conflicts with authorities. High-loading items included fear of deportation (PMLD24), asylum process delays (PMLD7), BAMF interview (PMLD22), conflicts with BAMF officials (PMLD23), and discrimination (PMLD9).

The second factor captured structural and financial hardship, such as poverty, difficulties finding work, precarious housing, and lack of institutional or charitable support. Key items included poverty/lack of basic needs (PMLD12), poor working conditions (PMLD11), cannot find work (PMLD10), little government support (PMLD5), little charitable support (PMLD6), and housing situation (PMLD20).

The third factor represented health and service access, particularly challenges in obtaining appropriate medical or psychological help. High-loading items included no treatment for health problems (PMLD1), poor access to emergency care (PMLD2), poor access to long-term care (PMLD3), poor access to counseling (PMLD4), and language problems (PMLD8).

The fourth factor described emotional and family-related strain, including separation from family, relational disruptions, and feelings of social isolation. Key items included separation from family members (PMLD14), homesickness (PMLD19), isolation (PMLD18), loneliness/boredom (PMLD17), and lack of emotional support (PMLD25).

Most items loaded clearly on a single factor (≥ 0.30), supporting simple structure. A few items (e.g., no access to traditional food (PMLD21), no access to religion (PMLD26), and climate difficulties (PMLD27)) did not show meaningful loadings and were excluded from interpretation. The average item complexity was 1.6, indicating limited cross-loading.

These results support a multidimensional conceptualization of PMLDs, encompassing distinct but interrelated domains of legal, economic, health-related, and relational stressors. The full pattern of factor loadings is provided as supplementary material [see Additional file 2].

### Association between post-migration stressors and depressive symptoms

To examine the relationship between post-migration stressors and depressive symptoms, we conducted a linear regression model with total PMLD scores predicting PHQ-9 scores, including interaction terms for latent class membership. The overall model was significant (R² = 0.20, F(5, 135) = 6.76, *p* <.001). However, the interaction terms between PMLD burden and class membership were not statistically significant (all ps > 0.19), indicating that the strength of the association did not differ significantly across classes. However, separate Pearson correlations within each class revealed a more nuanced pattern. In Class 3 (*Low Burden*), PMLD and depressive symptoms were moderately correlated (*r* =.36, *p* =.005), and a similar correlation was observed in Class 2 (*r* =.34, *p* =.019). In contrast, the correlation was small and non-significant in Class 1 (*r* =.12, *p* =.47). These findings indicate that the link between post-migration difficulties and depression may be more pronounced in individuals reporting moderate or low overall stress burden, potentially pointing to different mechanisms of distress across classes.

### Network analytic perspective of association between depression and PMLD

**Fig. 2:**
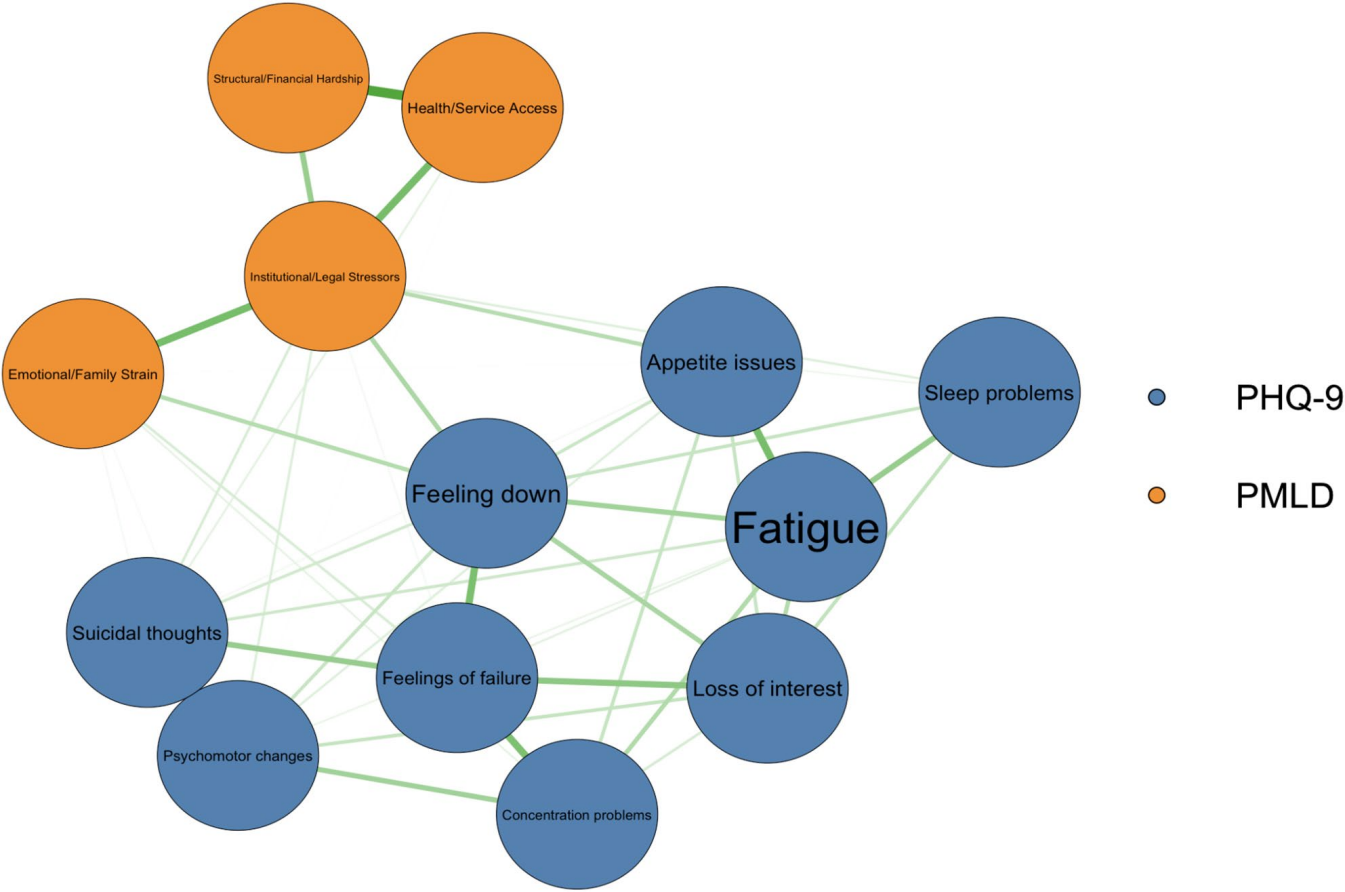
Network of depression symptoms and PMLD factors.The network illustrates the partial correlations between depressive symptoms and post-migration living difficulties (PMLD). Nodes represent individual symptoms (blue) and PMLD factors (orange). Edges reflect the strength of the associations after controlling for all other variables in the network. Thicker edges represent stronger associations. The network was estimated using regularized partial correlation (EBICglasso) and visualized with the Fruchterman-Reingold layout. PMLD = Post-Migration Living Difficulties; PHQ-9 = Patient Health Questionnaire-9

We estimated a network including nine depressive symptoms (PHQ-9) and four post-migration living difficulty (PMLD) factors to explore the structure of symptom-environment interactions. The resulting network highlights associations both within and between these two domains (Fig. 2).

**Fig. 3:**
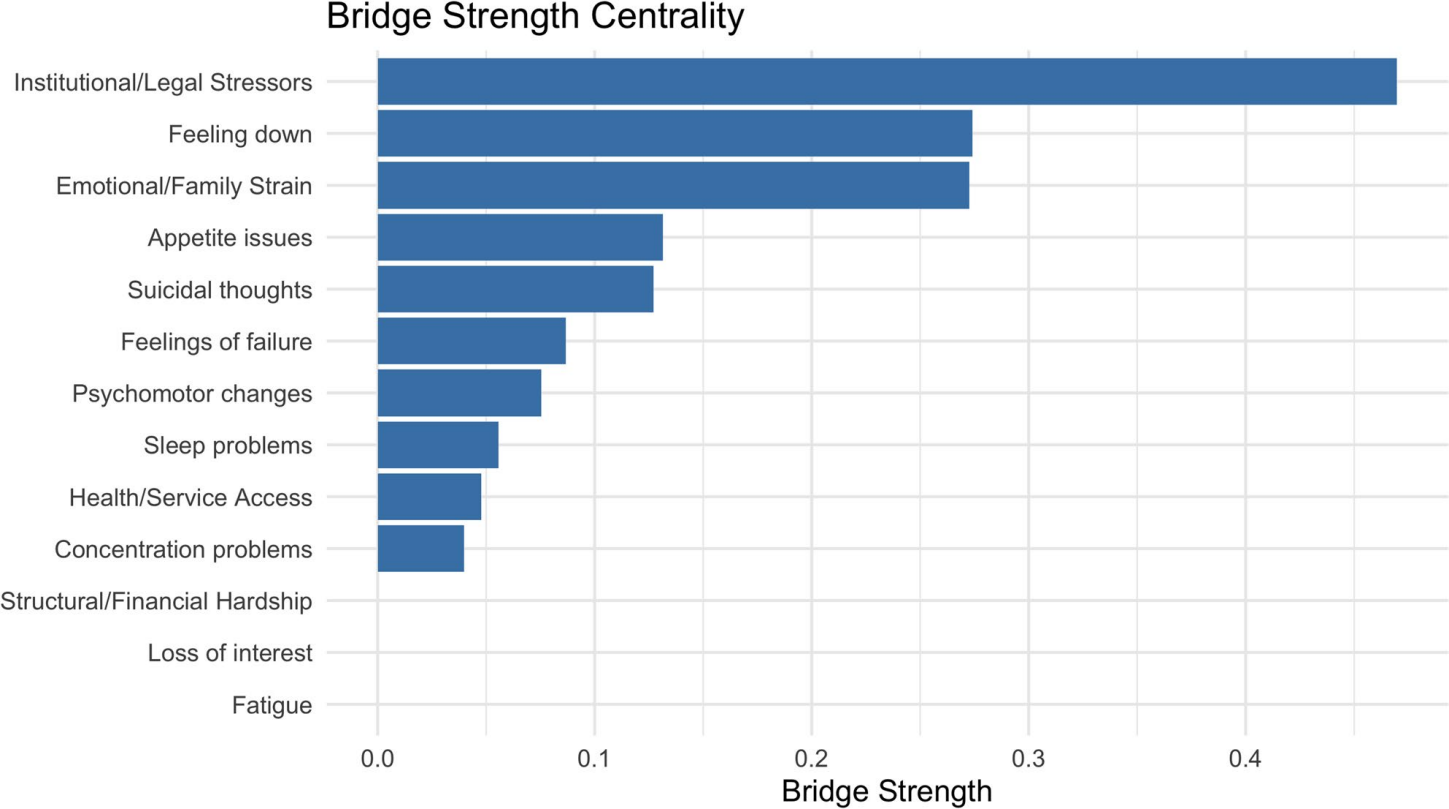
Bridge strength centrality for depression symptoms and PMLD factors**.** Horizontal bar chart showing bridge strength centrality values for nodes in the network including depressive symptoms (PHQ-9 items) and post-migration living difficulty (PMLD) factors. Higher values indicate greater connectivity between communities (i.e., depression and PMLDs)

To identify which nodes function as key connectors between PMLDs and depressive symptoms, we computed bridge strength centrality (Fig. 3). Bridge strength centrality quantifies the extent to which a given node connects to nodes in other communities—in this case, the depression and PMLD domains.

The node with the highest bridge centrality was Institutional/Legal Stressors (Bridge strength centrality = 0.47), suggesting that this factor acts as a crucial link between depressive symptoms and broader structural post-migration challenges. Notably, the PHQ-9 symptom *“Feeling down”* (PHQ9_2) also exhibited substantial bridge strength centrality (0.27), followed closely by the *Emotional/Family Strain* factor (0.27). Other PHQ-9 symptoms such as *Appetite issues* (PHQ9_5; 0.13) and *Suicidal thoughts* (PHQ9_9; 0.13) also contributed to bridging across communities, albeit to a lesser extent.

By contrast, some nodes (e.g., *Loss of interest*, *Fatigue*, and *Structural/Financial Hardship*) showed negligible or zero bridge strength centrality, indicating a more isolated or within-community role in the network. These findings suggest that *legal and institutional difficulties*—such as fear of deportation or restricted legal rights—and *emotional strain related to family separation* may be particularly important pathways linking environmental stressors to depressive symptoms among refugees.

## Discussion

This study set out to examine how different patterns of PMLDs affect depressive symptoms among refugees and asylum seekers. Rather than treating stressors as a uniform burden, we aimed to identify distinct stressor profiles, assess their relationship with depression, and explore how specific stress factors act as bridges between environmental adversity and psychological symptoms.

Using a combination of latent profile and network analyses, we identified three qualitatively distinct stressor profiles with varying levels of depressive symptomatology. To capture the underlying dimensions of post-migration stress, we first conducted an exploratory factor analysis, which revealed four core domains: institutional/legal difficulties, structural hardship, health/service access, and emotional/family-related strain. These factor-derived domains—rather than the latent profiles—were then included in the network analysis to examine their dynamic associations with depressive symptoms. Institutional/legal difficulties and emotional/family-related strain emerged as central bridges linking environmental adversity to psychological distress. These findings offer a more nuanced understanding of how specific stressor domains contribute to mental health outcomes in displaced populations. Legal status may shape PMLDs differently, especially institutional/legal stressors, so our pooled findings should be interpreted across both refugees and asylum seekers.

First, latent profile analysis revealed three distinct subgroups with differing PMLD exposure. Class 1 – High post-migration stressors showed the highest overall burden, Class 2 – Moderate post-migration stressors (predominantly family-related) experienced pronounced relational distress, and Class 3 – Low post-migration stressors reported relatively few difficulties. These profiles align with previous research emphasizing heterogeneity in post-migration stressors [5, 35] and extend findings by Byrow et al. [19], who also identified distinct latent classes among refugees based on PMLD exposure. Importantly, gender was not a significant factor in determining class membership or depressive symptom severity, suggesting that the identified patterns may operate similarly across male and female participants in this clinical sample. Nonetheless, it remains crucial to consider gender in the interpretation of our findings. Prior research consistently shows higher prevalence of depression and anxiety among women in refugee populations, often linked to relational stressors and traditional gender roles. In contexts such as Afghanistan, restrictive norms around women’s mobility, education, and social participation may exacerbate post-migration stressors, particularly in domains such as isolation, family separation, or limited access to services. Recent studies confirm that Afghan women report higher levels of depression, anxiety, and suicidal ideation compared to men [36–39]. The absence of significant gender effects in our study may therefore reflect the high overall clinical burden across participants, which may have overshadowed more subtle gender-related differences. Unlike their community-based sample, our study examined a clinically referred population, thereby underscoring the relevance of stressor profiles in more severely affected individuals.

Second, exploratory factor analysis confirmed that PMLDs are multidimensional, comprising four interrelated domains: institutional and legal stressors (e.g., fear of deportation, asylum uncertainty), structural and financial hardship (e.g., poverty, job insecurity), health and service access (e.g., limited access to medical or psychological care), and emotional and family-related strain (e.g., loneliness, family separation). These dimensions reflect both systemic conditions and intimate relational disruptions, echoing ecological models of refugee distress [6, 40]. Importantly, our factor structure closely mirrors that of Byrow et al. [19], who similarly identified legal, economic, and relational domains, thereby supporting the construct validity of these stressor categories across refugee contexts.

Third, while total PMLD burden was positively associated with depression, this link varied by group. In the low and moderate burden classes, higher stressors predicted greater depressive symptoms, but in the high burden group, the relationship was weaker. This may reflect stress saturation effects [12], where chronic adversity blunts emotional reactivity or limits perceived agency. This nonlinear pattern was not observed in previous LPA studies (e.g [19]).,. One possible explanation lies in fundamental differences between the samples. Byrow and colleagues [19] explicitly excluded individuals residing in detention or immigration facilities, thereby focusing on refugees who were already integrated into the Australian community and had a secure legal status. As such, their sample likely experienced lower levels of chronic adversity compared to our participants, many of whom were still exposed to ongoing legal uncertainty, restricted housing conditions, and limited access to services. In contrast, our sample consisted of treatment-seeking individuals in Germany, many of whom were still in asylum procedures or living in communal refugee accommodations under highly uncertain conditions. These contextual differences—particularly regarding legal security and housing situation—likely shaped not only the level of post-migration stressors but also how these translated into depressive symptoms. This suggests that buffering mechanisms and psychological responses to adversity may differ substantially between community-based and clinically referred refugee populations.

Finally, network analysis identified Institutional/Legal Stressors as the most central bridge connecting stress to symptoms, followed by Emotional/Family Strain. These findings support previous studies showing that asylum insecurity and family separation are among the most potent drivers of mental health decline in displaced populations [2, 15]. The symptom “feeling down” (PHQ-9 item 2) also emerged as a central bridge node. Its role may reflect not only transdiagnostic relevance, but also cultural expressions of distress, such as the idiom “thinking too much” frequently reported among refugee populations [41].

In contrast, structural hardship and symptoms like fatigue showed limited bridge centrality, suggesting they may function more as downstream outcomes than active connectors. This insight adds to the growing application of network analysis in refugee mental health [40, 42] and demonstrates the value of identifying “bridge symptoms” for targeted intervention. Overall, the network findings underscore that not all stressors or symptoms equally drive psychological distress—highlighting the importance of targeting central nodes, such as legal insecurity and low mood, in both assessment and intervention.

Together, these findings highlight the central role of post-migration living difficulties (PMLDs) as key drivers of psychological distress in refugee populations. Notably, our results suggest that certain domains—particularly legal insecurity and family-related separation—may be especially central in maintaining psychological distress, and their neglect could potentially hinder treatment effectiveness. While interventions targeting specific stress-symptom “bridges” such as asylum insecurity and family separation may yield therapeutic benefits, it is important to recognize that PMLDs reflect broader socio-political determinants that extend beyond individual-level treatment. Addressing these complex challenges requires a multiprofessional approach integrating legal aid, social support services, and community-based resources alongside culturally sensitive mental health care [3, 12].

Moreover, it is crucial to acknowledge that certain PMLDs are amenable to immediate resolution within clinical settings. Many stressors stem from systemic issues such as protracted asylum procedures, discrimination, or restricted family reunification policies, which fundamentally require political and structural change [1, 3]. Consequently, mental health interventions should be embedded within a framework that fosters advocacy and collaboration across healthcare, legal, and social sectors. At a broader policy level, expanding safe legal pathways for asylum and family reunification could directly reduce two of the most central stressors identified in this study: legal insecurity and prolonged family separation. Providing faster and more transparent asylum procedures, access to community-based housing, and early integration programs may further alleviate structural and relational burdens. Embedding such measures alongside equal access to healthcare could represent an important step toward reducing the mental health burden among forcibly displaced populations.

### Limitations

We pooled refugees and asylum seekers and lacked detailed asylum-trajectory data; future work should examine status-specific pathways. While the study provides novel insights into the structure and clinical relevance of post-migration living difficulties, several limitations should be noted. First, asylum-related legal factors—such as current residency status, stage and duration of the asylum procedure—were not systematically assessed. Given prior evidence that legal insecurity significantly shapes mental health outcomes, future studies should incorporate these variables to better contextualize stressor exposure and subgroup membership.

Second, although the multicentre design enhances generalizability, site effects were not formally examined. As such, it remains unclear whether regional or institutional differences may have influenced the observed profiles. Given the exploratory nature of this analysis and the sample size, including site-level variables would have added substantial complexity and reduced statistical power. Future studies with larger cohorts should address this question more systematically.

Third, while participants were recruited from clinical settings, variability in service access, housing conditions (e.g., communal vs. private accommodation), and cultural framing of distress may have influenced how post-migration stressors were subjectively perceived or communicated. These contextual factors may shape symptom expression and should be explored in more detail in future studies.

Moreover, although emotional and family-related strain emerged as a central stressor domain, data on whether participants fled alone, with family or on family status more broadly (e.g., being a caregiver or parent) were not collected. This limits interpretation of separation-related stress and should be addressed in future studies.

Future research should also examine how broader contextual factors—including asylum trajectory, length of stay in host countries, arrival cohorts, and country of origin—modulate the impact of post-migration living difficulties on mental health outcomes. Longitudinal and mixed-methods designs will be especially valuable to trace how such stressors become internalized and to identify culturally specific resilience mechanisms.

## Conclusion

This study underscores the central role of PMLDs in shaping the mental health of refugees and asylum seekers. Using latent profile and network analysis, we identified distinct patterns of post-migration stressors and their differential associations with depressive symptoms. In particular, institutional/legal challenges and emotional/family-related strain emerged as key drivers and bridging mechanisms of psychological distress.

However, given the absence of data on whether individuals fled alone or with family members, conclusions regarding the nature and intensity of separation-related strain should be drawn with caution.

The findings underscore the need for mental health interventions that extend beyond symptom management to address structural and relational stressors. Sustainable recovery for displaced populations requires that clinical efforts be embedded within a broader socio-political framework that promotes legal security, family reunification, and social inclusion.

## Supplementary Information

Supplementary Material 1.Case-dropping bootstrap analysis of node strength

Supplementary Material 2.Factor loadings from exploratory factor analysis of PMLD items

## Data Availability

The datasets generated and/or analysed during the current study are not publicly available due to confidentiality and ethical considerations related to the participants’ refugee status. However, the data are available from the corresponding author upon reasonable request.

## Abbreviations

PHQ-9: Patient Health Questionnaire-9
PMLD: Post-Migration Living Difficulties
LPA: Latent Profile Analysis
EFA: Exploratory Factor Analysis
RCT: Randomized Controlled Trial
DSM-5: Diagnostic and Statistical Manual of Mental Disorders, Fifth Edition
KMO: Kaiser-Meyer-Olkin
RMSEA: Root Mean Square Error of Approximation
TLI: Tucker-Lewis Index
