# Supplementary figures and images for: Carrying what came after: post-migration difficulties and depression among refugees and asylum seekers
Source: Confl Health. 2025 Dec 2;19:86. doi: 10.1186/s13031-025-00728-3 (PMC12670758; doi:10.1186/s13031-025-00728-3)

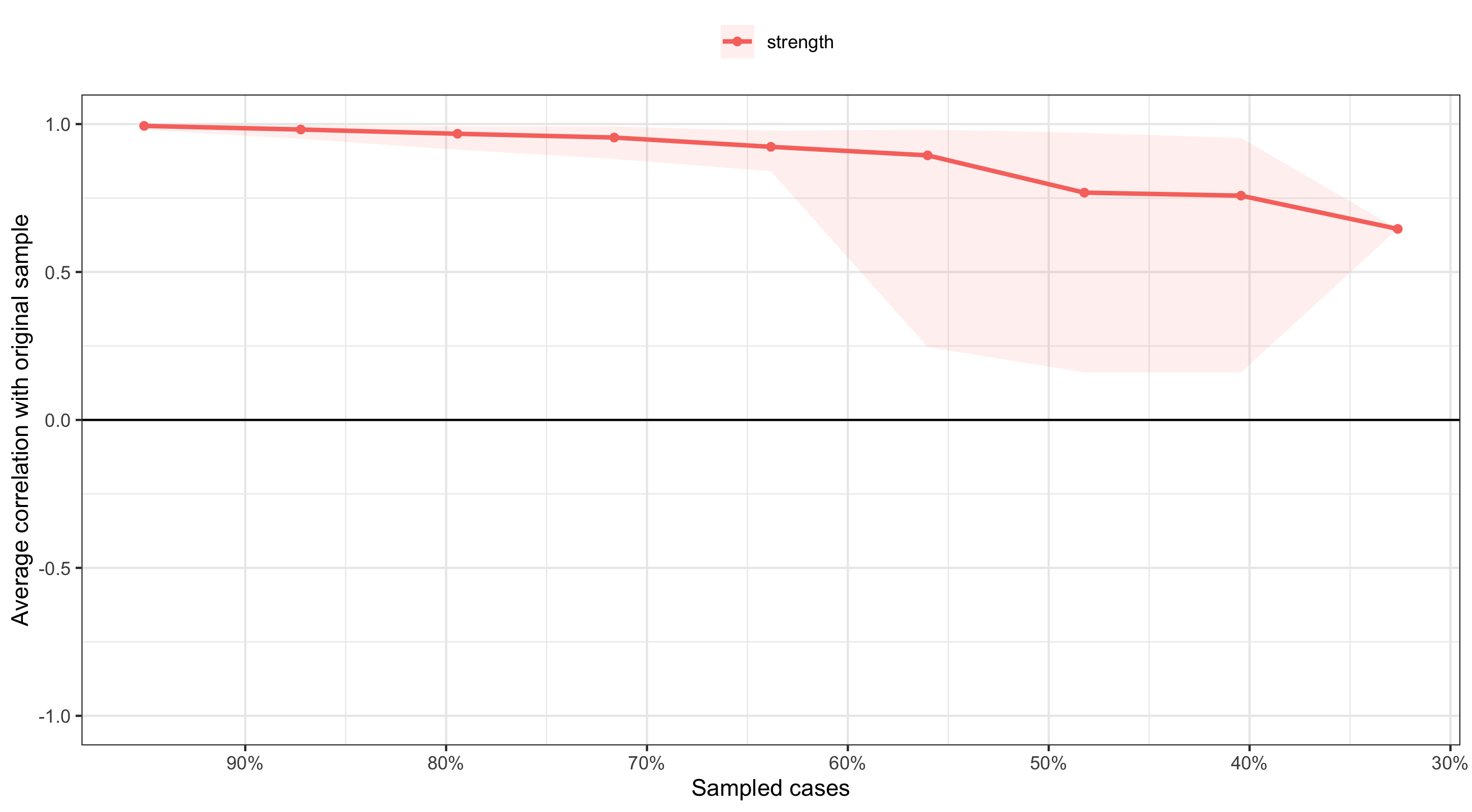

Supplement: Supplementary file 1 — Supplementary Material 1.Case-dropping bootstrap analysis of node strength [file 13031_2025_728_MOESM1_ESM.png]

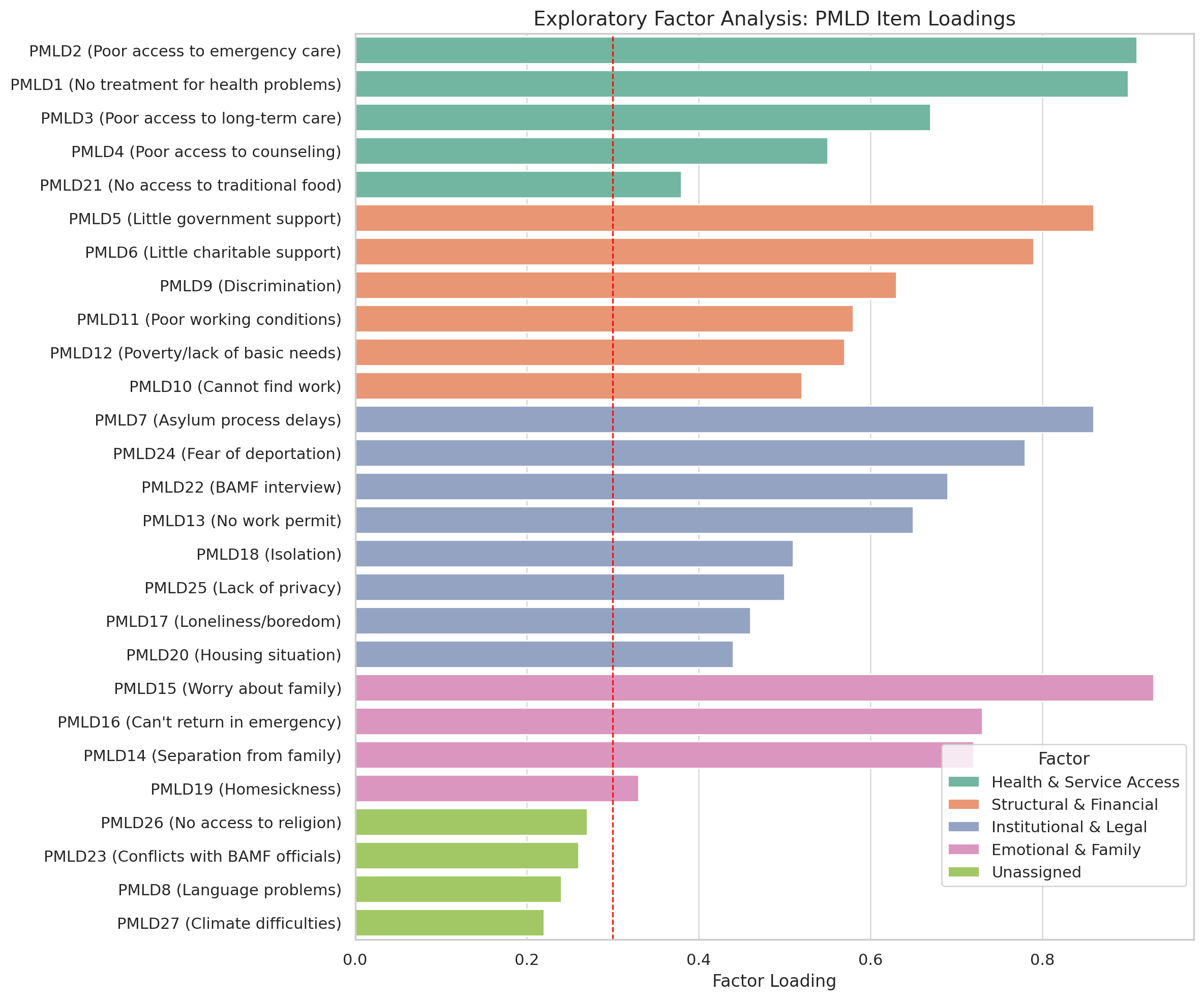

Supplement: Supplementary file 2 — Supplementary Material 2.Factor loadings from exploratory factor analysis of PMLD items [file 13031_2025_728_MOESM2_ESM.png]
